# X-ray and nuclear spectroscopies to reveal the element specific oxidation states and electronic spin states for nanoparticulated manganese cyanidoferrates and analogs

**DOI:** 10.3390/physchem4010003

**Published:** 2023-12-25

**Authors:** Hongxin Wang, Songping D. Huang, Anthony T. Young, Stephen P. Cramer, Yoshitaka Yoda, Lei Li

**Affiliations:** 1 SETI Institute, Mountain View, CA 94043, United States; 2 Department of Chemistry and Biochemistry, Kent State University, Kent, OH 44242, United States; 3 Lawrence Berkeley National Laboratory, Berkeley, CA 94720; 4 Precision Spectroscopy Division, SPring-8/JASRI, Sayo, Hyogo 679-5198, Japan; 5 Synchrotron Radiation Research Center, HSTA, Kouto, Hyogo, 679-5165, Japan

**Keywords:** nanoparticulate manganese cyanoferrates, non-gadolinium magnetic resonant imaging agents, L-edge X-ray absorption spectroscopy, L XAS, nuclear resonant vibrational spectroscopy, NRVS, oxidation states, electronic spin states

## Abstract

In this publication, the potential non-gadolinium magnetic resonant imaging agent – the nanoparticulate K_2_Mn[Fe(CN)_6_], its comparison sample KFe[Co(CN)_6_], as well as their reference samples were measured and analyzed with Mn, Co and Fe L-edge X-ray absorption spectroscopy (L XAS). From the information obtained, we conclude that K_2_Mn[Fe (CN)_6_] has a high spin (hs)-Mn(II) and a low spin (ls)-Fe(II), while KFe[Co(CN)_6_] has a hs-Fe(II) and a ls-Co(III). In these Prussian blue (PB) analog structures, L XAS also concluded that the hs-Mn(II) in K_2_Mn[Fe(CN)_6_] or the hs-Fe(II) in KFe[Co(CN)_6_] is bonding to the N in the [M(CN)_6_]^4−/3−^ ions [where M = Fe(II) or Co(III)], while the ls-Fe(II) in K_2_Mn[Fe(CN)_6_] or the ls-Co(III) in KFe[Co(CN)_6_] is bonding to the C in the [M(CN)_6_]^4−/3−^ ion, suggesting the complexed metalloligand [Mn(II) or Fe(II)] occupy the N-bound site in PB. Then, nuclear resonant vibrational spectroscopy (NRVS) was used to confirm the results from the L XAS measurements: the Mn(II), Eu(III), Gd(III), Fe(II) cations complexed by [M(CN)_6_]^n−^-metalloligand are all taking the N-bound site in the PB-like structures. Our NRVS studies also approve that iron in K_2_Mn[Fe(CN)_6_] compound has 2+ oxidation state and is surrounding by the C donor atoms in the [M(CN)_6_]^n−^ ions.

## 1. Introduction

**Figure 1.**
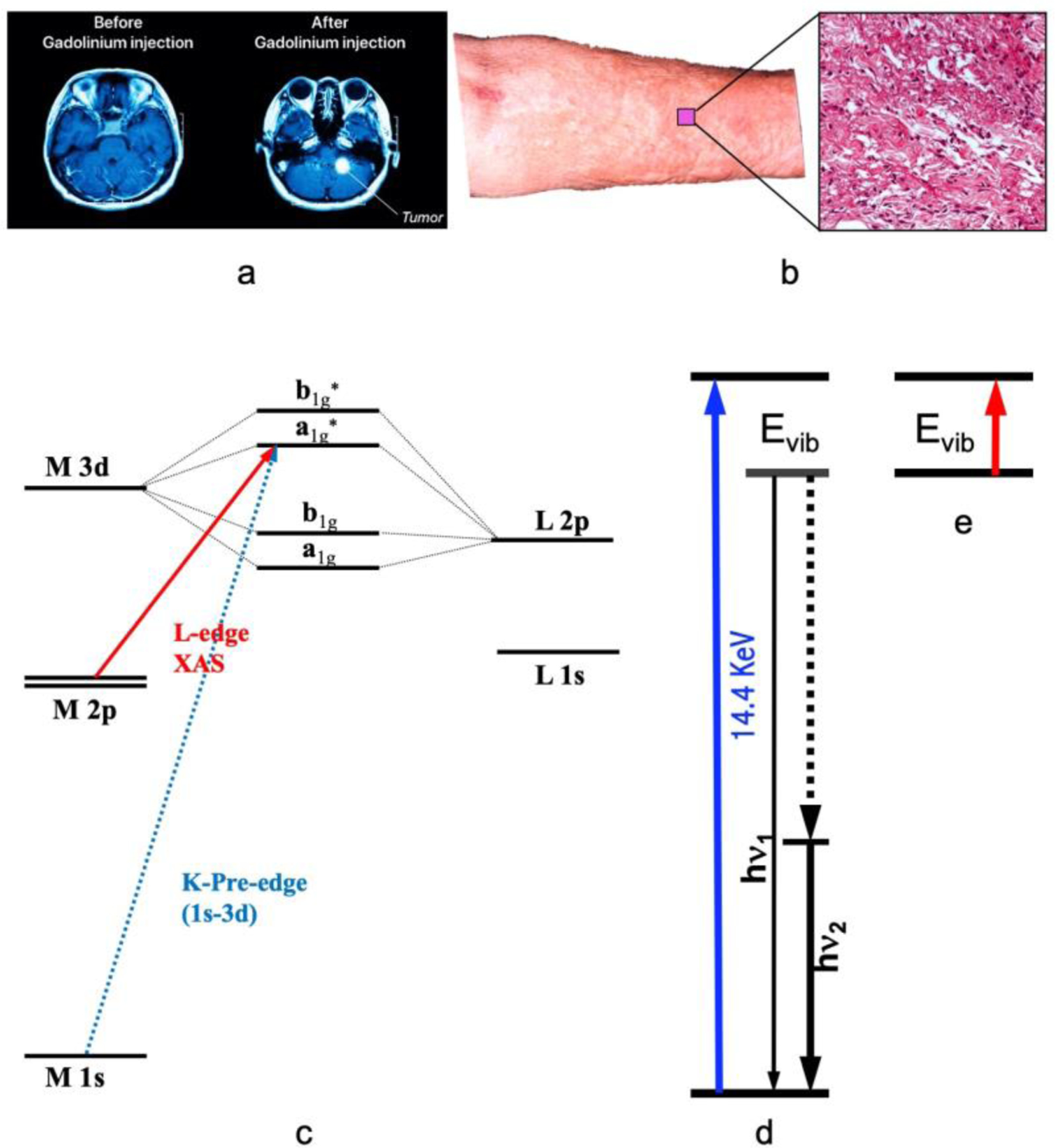
(**a**) The illustrative MRI images without (left) vs. with (right) a contrast agent; (**b**) the skin surface and its microscopic profile of NFS; (**c**) illustrative K- and L-edge X-ray absorption spectroscopy (XAS) transitions and the associated energy levels; (**d**) nuclear resonant vibrational spectroscopy (NRVS) transitions which measures the vibrations indirectly but with many advantages; (**e**) infrared absorption spectroscopy (IR), which measures vibrations directly.

Magnetic resonance imaging (MRI) has emerged as a prominent non-invasive and nonradioactive tool for diagnosing various diseases and/or testing human organ functions [1, 2]. To aid the diagnoses, it is often necessary to administer an imaging agent to improve the contrast [1, 3]. This is especially true for imaging the brain or spine where small details are often pursued [4]. For example, Figure 1(a) illustrates the use of a contrast agent for the MRI of the brain. Specifically, the right-sided Figure 1(a) shows a much clearer and more detailed picture with the use of a contrast agent in comparison with the one on the left where no contrast agent is used. The current clinical MRI agents all contain a single paramagnetic metal ion in their centre, with gadolinium (Gd) is the most frequently used metal so far in the commercial contrast agents. e.g., Gd-DTPA (Magnevist^®^, Schering AG, Germany). These Gd based agents are used in about 1 in 3 of MRI scans to improve the clarity of the images for the internal structures of the human body [4–6]. Often, use of an MRI contrast agent improves the visibility of inflammation, tumours, and blood vessels, or, in some cases, even blood flow or the real time function of blood vessels. In turn, a better MRI image provides a better medical diagnostic accuracy. For example, many detailed heart or brain abnormalities can well be assessed [4, 6].

Although Gd contrast agents have proven to be invaluable, they are not without drawbacks. In extreme cases, these agents can lead to a serious disease called nephrogenic systemic fibrosis (NSF) [6–11]. An example of NFS patients’ skin appearance as well as the detailed looking are shown as in Figure 1(b) and its right insert. These are the Gd-related deposits. In 2010, synchrotron radiation (SR)-based X-ray fluorescence microscopy (SXRF) and extended X-ray absorption fine structure spectroscopy (EXAFS) were used to chemically and spectroscopically characterize the Gd deposits in the skin from an NSF patient [8]. It has been concluded that the Gd deposits in that NSF case consist of a Gd-phosphate material with almost no Gd remaining coordinated to the original organic chelator [8]. One reason is that Gd^3+^ ion has a very similar radius with that of Ca^2+^ (i.e., 1.07 Å for Gd^3+^ and 1.02 Å for Ca^2+^) and can replace the Ca^2+^ in human body and skin [8]. Therefore, Gd-DTPA or other Gd-based agents are still considered harmful in general [6–9, 11–14], and hence safer alternative imagining contrast agents need to be explored.

From the entire periodic table, there are only a few elements that are considered suitable as MRI enhancement candidates. Specifically, they need to have a high number of unpaired electrons, a long electronic relaxation time, and a stable oxidation state under the physiological conditions inside the human body at the same time. Hence, the suitable candidates include, for example, Gd(III) (S=7/2), Eu(II) (S=7/2), Fe(III) (S=5/2), and Mn(II) (S=5/2). Among these, Mn(II) ion is a bio-essential element and thus more compatible with biological cells than Gd(III) is. It also has a high electronic spin (S=5/2) and a fast rate in water exchange, possessing almost ideal physical, chemical, and biological properties for MRI applications [15, 16]. These properties led to two U.S. Food and Drug Administration (FDA)-approved contrast agents for clinical use, i.e., MnDPDP (DPDP = dipyridoxyl diphosphate; Teslascan^®^) and MnCl_2_ (LumenHance^®^). However, all Mn complexes including MnDPDP are kinetically labile and susceptible to the interaction with other metals such as Zn, Cu and Fe, etc. In particular, Mn(II) from these agents can be released from the contrast agents to the human body to cause problems, e.g., to replace Zn(II) [17, 18]. MnCl_2_ in aqueous solution can exist as [Mn(H_2_O)_6_]^2+^ and Cl^−^ ions in the stomach. Even in a strong HCl environment, it does not turn to form the suspected [MnCl_n_]^(n−2)−^ complexes, which is very different from CuCl or AgCl. Although Mn(II) has much less toxicity than Gd(III), absorption of excessive amount of Mn can still cause neurotoxicity. Other single metal centred coordination complexes also show various disadvantages and various potential toxic concerns.

These concerns may be addressed by incorporating Mn (or other proper metal ions including Gd) into the Prussian blue (PB) structure in the form of nanomaterials [19, 20]. PB has an extremely high formation constants (e.g., K_f_ = 1.6×10^31^ for PB vs. 10^23^ for Gd-DTPA) and therefore is much safer for medical use. For example, it is one of the most important medications on the World Health Organization’s List of Essential Medicines [21]. In general, it has a chemical formula of Fe^III^_4_[Fe^II^ (CN)_6_]_3_·xH_2_O, with a structure shown in the Figure 1(b) in [22]. For an easier reference, we copied it as Figure S1 in the Supporting Information (SI). A quarter of Fe(II) centres along with CN^−^ ions have to be absent from the crystal lattice in order to maintain a ratio of Fe(III):[Fe^II^(CN)_6_]^4−^ = 4:3 to realize the electrical neutrality of PB [23]. This arrangement creates void inside the structure that is filled by a varying number of H_2_O molecules (i.e., x = 1 to 12). For PB–like analogs such as the manganese cyanidoferrates K_2_Mn[Fe(CN)_6_] to be explored in this study, Mn:Fe=1 is maintained instead, while a counterion of K^+^, or sometimes other monovalent ions, such as Li^+^, Na^+^, K^+^ Rb^+^, Cs^+^, Tl^+^, or (NH_4_)^+^, can be used to maintain the electrical neutrality for the whole compound.

These PB–like compounds, e.g. K_2_Mn[Fe(CN)_6_], have attracted a great deal of attention as potential MRI contrast agents in the past ten years [19, 20] [24] because: 1) the extremely high formation constant leads to an extremely stable complex [19, 20]. This in turn contributes to a much reduced risk of exposing the patients to the metal ions; 2) these complexes have a long blood circulation time, which is desirable for detailed imaging studies [25]; and 3) a given nanoparticle can form a superparamagnetic domain. Such material can thus possess a much higher magnetic susceptibility as compared with the paramagnetic materials composed of single molecules. This property can produce a much higher MRI sensitivity. On the other hand, nanoparticles are still small enough to be freely transported inside the human bloodstream.

There are a few major scientific issues concerning K_2_Mn[Fe(CN)_6_] in general: 1) what are the oxidation states of the two metals inside the complex?, i.e. whether this complex has a Mn(II)/Fe(II), a Mn(II)/Fe(III), a Mn(III)/Fe(II) or a Mn(III)/Fe(III); 2) what are the electronic spin states (i.e., ls vs. hs, where ls = low spin and hs = high spin) of the two metal sites? 3) in PB, the ls-Fe(II) is taking the C-bound position in the [Fe(CN)_6_]^4−^ ion and the hs-Fe(III) is taking the N bound position to the [Fe(CN)_6_]^4−^ ion. In K_2_Mn[Fe(CN)_6_], whether the Mn takes the positions for the original N-bound hs-Fe(III) sites or those for the C-bound ls-Fe(II) sites in comparison with PB in Figure S1? For such simple complex, the chemical intuition seems able to suggest that the tightly bound [Fe(CN)_6_]^4−^ ions from the starting material of K_4_[Fe(CN)_6_] will remain its original Fe(II) (S=0), while the complexed Mn can take PB’s hs-Fe(III) position and in principle be in different oxidation states [e.g., Mn(III) vs. Mn(II)] and/or different spin states (hs vs. ls) according to the Mn source. As the C-bound ls-Fe(II) in [Fe(CN)_6_]^4−^ ion has an S=0, the total spin in such PB-like complex has the electronic spin of the N-bound metal alone – for instance, from the hs-Mn(II), S=5/2. Nevertheless, it appears to be very meaningful to use modern spectroscopies [26–35] to shed some lights directly on these issues which are also critical for evaluating its suitability as an MRI agent [19, 20].

SR-based X-ray absorption spectroscopy [abbreviated as XAS hereafters, Figure 1(c)] measures the electronic transition from a core shell to a valence shell. As XAS is sensitive to valence electrons and it is element-specific (related to core shell) at the same time, it is one of the best methods to investigate the oxidation state of a specific element in different chemical compounds or enzymes [27, 29–32, 36, 37]. For the 3d transition metal ions (e.g., Mn, Co or Fe ion), K-edge XAS uses a hard X-ray beam of > 4000 eV to study the transitions of 1s → 3d, → 4p and → continuum [Figure 1(c), the transition designated by the dashed blue line]. Although the information obtained from the K-edge XAS seems comprehensive [27, 32–34, 37], L-edge XAS [29–31, 36, 38, 39] [Figure 1(c), the transition designated by the solid red line] as well as L-edge RIXS or L-edge X-ray emission spectroscopy[40–43] have several advantages over the K-edge XAS for studying electronic structures such as oxidation states and spin states. These advantages include a direct probe of the ligand-metal bonding orbital (3d); a dipole-allowed 2p → 3d transition (vs. 1s → 3d in K-edge XAS); a better energy resolution (e.g. 0.1 eV vs. 1 eV); and a rich spectral multiplet which is specific to particular electronic structures and coordination environments [29–31, 36, 38, 39].

Nuclear resonant vibrational spectroscopy (or NRVS for short) is another SR-based modern spectroscopic technique that was widely used by physicists, chemists, biochemists, and materials scientists. It measures the phonons, or, in other words, vibrational modes associated with the nuclear transition for a specific isotope, as illustrated in Figure 1(d). The most frequently used NRVS to date is the ^57^Fe NRVS which has a nuclear resonant transition at 14.4 keV [28, 44–49]. In short, it is a scattering spectroscopy in general and a nuclear resonant scattering spectroscopy in particular. In comparison with the conventional infrared absorption spectroscopy (IR) [50, 51] [Figure 1(e)], it measures vibrations indirectly but has several distinct advantages. The most prominent advantages include but not limited to being isotope (e.g., ^57^Fe) specific for studying complicated systems [28, 48, 49, 52–55], having an almost zero background [28, 48], and being able to obtain a theoretically calculable partial vibrational density of state (PVDOS) [28, 45–47, 56]. These advantages make it a better method in comparison with the laboratory-based IR [28] [50, 51], Raman [28] and laser induced fluorescence (LIF) spectroscopies [57–59] as well as SR-based inelastic X-ray scattering (IXS) [28] [60]. This modern spectroscopy became available in mid 1990s due to the development of the third-generation SR sources which provides the strong X-ray pulses with specific timing structure, advanced X-ray optics which lead to a monochromator with an 1 meV energy resolution to measure vibrations, and modern detectors which extract weak nuclear scattering signal from the huge electronic scattering counts in the time domain, and in turn, it has pushed a great advancement in physics, chemistry, biochemistry and materials science, etc. for the past 28 years [45–49, 55]. In chemistry and bioinorganic chemistry, for example, this technique has uncovered Fe-S/P/Cl, Fe-CO/CN/NO and Fe-H/D vibrational modes inside various inorganic complexes as well as dilute iron enzymes [28, 61–64], and thus become an excellent pinpointing tool to study iron-specific electronic and structural properties, including iron oxidation state(s) and coordination environment. For PB–like compounds, it can pinpoint to the features attributable to the N-blund hs-Fe(III) or those to the C-bound ls-Fe(II) [22, 28].

In this publication, the K_2_Mn[Fe(CN)_6_] complex was first measured and analyzed with the Mn and Fe L-edge XAS. For comparison, KFe[Co(CN)_6_] as well as their reference samples (K_4_[Fe(CN)_6_] and K_3_[Co(CN)_6_]) were also measured and analyzed with the Fe and Co L-edge XAS. From the information obtained from these L-edge XAS measurements, in reference to Figure S1, K_2_Mn[Fe(CN)_6_] is confirmed to have a hs-Mn(II) bound to the [Fe(CN)_6_]^4−^ ion in the N position in 6CN^−^ and a ls-Fe(II) bound to the 6CN^−^ in the C position. In comparison, KFe[Co(CN)_6_] has a hs-Fe(II) bound to the in the N position and a ls-Co(III) bound to the C position. For NRVS, ^57^Fe labelled (NH_4_)_2_Mg[^57^Fe(CN)_6_] and K^57^Fe[Co(CN)_6_] were first presented as the two standards to illustrate the nature of the two “peaks” in the natural PB NRVS [22]. Then, non-labelled K_2_Mn[Fe(CN)_6_] was measured with regional NRVS where only the energy regions around the resonant peak and the Fe-CN vibrational peak were measured [22, 28]. For discussion, the previously measured regional spectra for KGd[Fe(CN)_6_] [65] and KEu[Fe(CN)_6_] [22] are also cited and compared with K_2_Mn[Fe(CN)_6_]. NRVS concluded that the nanoparticulated manganese cyanidoferrates K_2_Mn[Fe(CN)_6_] as well as KGd[Fe(CN)_6_][65] and KEu[Fe(CN)_6_] [22] have a ls-Fe(II) which occupies the C-bound position in the [Fe(CN)_6_]^4−^ ion while the complexed Fe in K^57^Fe[Co(CN)_6_] has a hs-Fe(II) which takes the N-bound position to the [Co(CN)_6_]^3−^ ion.

## 2. Experiments and Materials

### 2.1 L-edge XAS measurements

**Figure 2.**
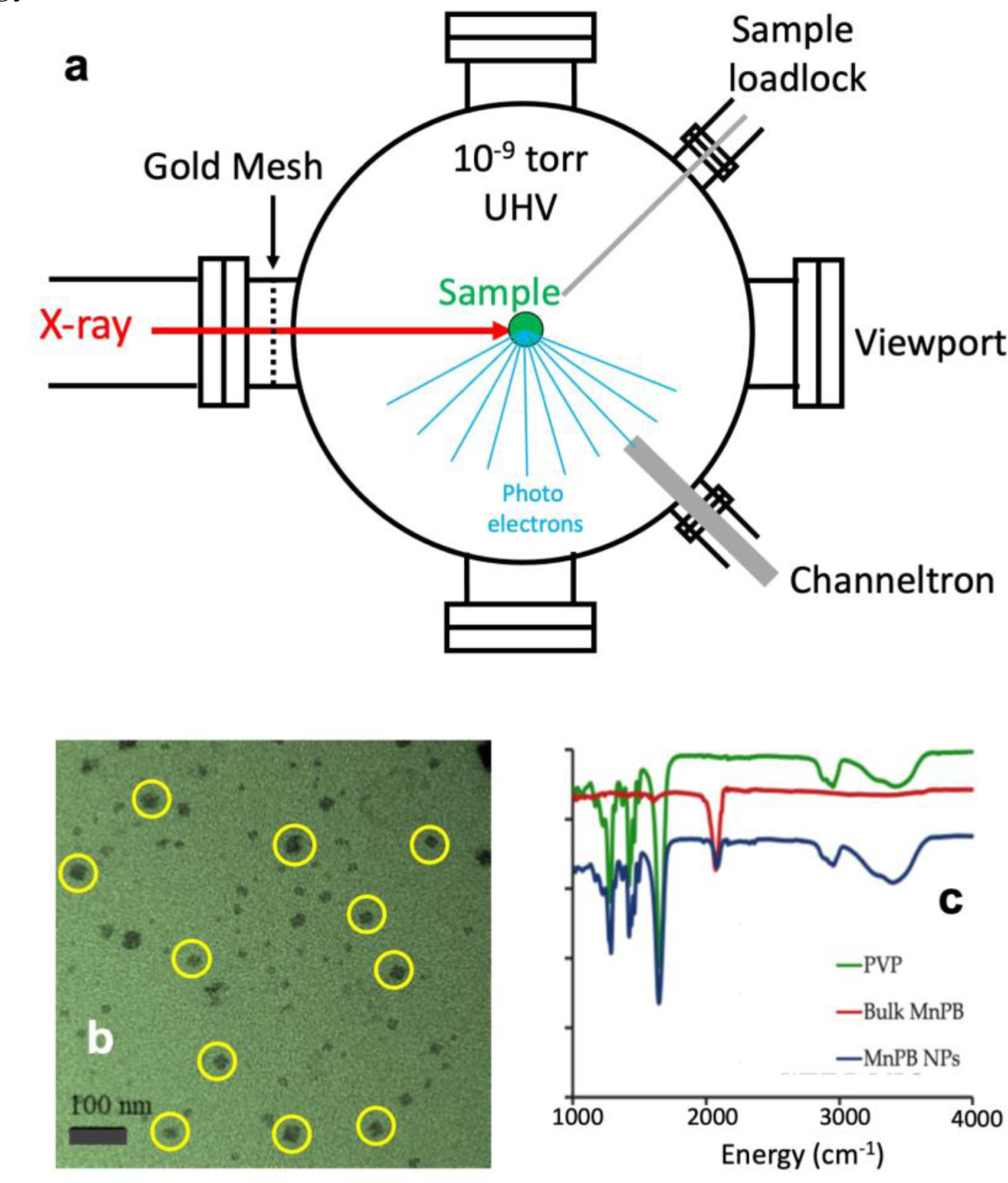
(**a**) A schematic drawing for 3d metal L-edge X-ray absorption spectroscopy (L XAS) measurement apparatus under ultrahigh vacuum (UHV); (**b**) a TEM image of the main sample - polyvinylpyrrolidone (PVP) coated K_2_Mn[Fe(CN)_6_] nanoparticles (PVP-coated Mn-PB NPs for short). The yellow circles are used as an eye-guide; (**c**) IR spectra for PVP (green), bulk Mn-PB (red) and PVP-coated Mn-PB NPs (blue).

Iron (Fe), manganese (Mn) and cobalt (Co) L-edge XAS spectra (or L XAS) for K_2_Mn[Fe(CN)_6_] as well as for its comparison samples, reference samples or energy calibration samples were measured at the undulator beamline 4.0.2 at the Advanced Light Source (ALS) in Berkeley, California. As previous reported [29–31, 36, 38], powdered solid state samples were grounded and pressed onto an ultra-high vacuum (UHV) compatible double stick carbon tape on the sample holder at room temperature, and loaded into a UHV compatible measurement chamber as illustrated in Figure 2(a).

Although all the samples in this publication are not air sensitive, to better control the surface condition (due to the fact that L-edge XAS is extremely surface sensitive [36, 38], the samples were ground, spread and mounted inside a nitrogen atmosphere glovebox maintained at an oxygen level of 1 ppm or less and then loaded into the UHV chamber via a vacuum compatible loadlock [Figure 2(a), upper right corner]. While the X-ray beam scanned through the intended energy region(s), Fe or Mn or Co L-edge XAS spectra were recorded using a channeltron electron multiplier [29–31, 36, 38] [Figure 2(a), lower right corner] to detect the emitted total photoelectrons whose intensity is proportional to the amount of X-ray absorption in the corresponding samples. Such spectra were further normalized to the incident radiation intensity monitored by using the photocurrent from a gold mesh [[Figure 2(a), left side]. Energy calibration was performed with appropriate standards before and after each set of the measurements. We noticed that there is no absolute energy calibration standard in the previous reports, the following standards and energy positions are chosen in this publication: Fe L-edge calibrations used the lowest energy L_3_ peak of Fe_2_O_3_ at 707.8 eV, Mn L-edge calibrations used the peak with the highest intensity of MnF_2_ at 640.0 eV, while Co L-edge was calibrated to the first sharp peak of Co(II)O at 778.8 eV. To reduce the possible radiation damage, the monochromator’s slits are set to 5 μm to decrease the beam flux to the order of 10^11^ photons/s and such obtained X-ray beam was further de-focused to 1×1 mm^2^ in its cross-section size on the sample. The energy resolution is at 0.1 – 0.2 eV.

### 2.2 NRVS measurements

NRVS spectra were recorded using a published procedure [28, 48, 49, 55] at SPring-8 beamlines BL09XU [66] in Japan. A high heat load monochromator (HHLM) produced 14.4 keV radiation with ~1.0 eV energy resolution, and a high energy resolution monochromator (HRM) [with crystals of Ge(422) and Si(975)x2] [66–68] subsequently produced 14.4 keV radiation with 0.8 meV energy resolution, suitable for measuring vibrations. The beam flux was ~1.8×10^9^ photons/s at the time of the measurements. During NRVS measurements, the samples were maintained at a cryogenic temperature (e.g. 10K at the temperature sensor) using a liquid helium cryostat. However, the real sample temperatures [28] derived from anti-Stoke/Stoke intensity ratios from the NRVS spectra were found to be 40–60K instead. As illustrated in Figure 1(d), while an incident X-ray beam (the thick blue) scans through an interested energy region to cover the nuclear transition (e.g., 14.4 keV for ^57^Fe) and the associated vibrations (e.g. Fe-CN), the extremely narrow bandwidth for the nuclear back radiation hν_1_ (the thin black line) can be used as an excellent intrinsic “spectrometer” to “measure” the scattered energy in precision while the signal vs. background can be distinguished in the time domain, just like LIF does [57–59]. NRVS thus does not need a low-throughput (diffraction based) spectrometer for filtering the scattered beam and thus have a much higher photon in and photon out efficiency in comparison with IXS which also measures vibrations [28]. The total intensities collected from both the direct nuclear fluorescence at hν_1_ (the thin black line) and the internally converted electron K shell fluorescence at hν_2_ (the thick and short black line) were recorded with a 2×2 avalanche photodiode (APD) array [28, 55]. This signal vs. the vibrational energy (E_vib_) forms a raw NRVS spectrum. NRVS has many prominent advantages, including being able to be converted to a partial vibrational density of state (PVDOS). The raw NRVS → PVDOS conversion is obtained with PHOENIX software package or a web-based version of PHOENIX [28, 56]. The detailed information about the NRVS instruments, measurement and analysis are the same as those published widely [28, 45–49, 55, 56] and will not be repeated here.

### 2.3 Sample preparation and basic characterization

The main sample, polyvinylpyrrolidone (briefed as PVP, chemical formula = C_10_H_15_NO_3_) coated K_2_Mn[Fe(CN)_6_] nanoparticles (Mn-PB NPs for short), was prepared with a procedure as published earlier [19, 20] and described in brief in SI.2. The proposed structure for Mn-PB is similar with the PB shown in Figure S1 except its hs-Fe(III) sites are substituted with Mn and an Mn:Fe = 1:1 ratio is maintained while K^+^ was used to balance the extra negative charge. PB was used as a platform and PVP was used as a coating agent to prevent the Mn-PB nanoparticles from agglomerating and to improve its chemical stability and biological compatibility. The as-synthesized nanoparticles were dialyzed in a semi-permeable membrane in distilled water. Any unbound PVP should effectively be removed during this process. From a transmission electron microscopy (TEM) image as shown in Figure 2(b), the PVP-coated Mn-PB NPs are 5 – 20 nm in size and are well separated from one another. No chunks of PVP or mixture of PVP and PB were observed. Characterization and in vitro as well as in vivo MRI studies of the Mn- PB NPs are similar as the one reported previously [69].

The IR spectra of PVP coated Mn-PB NPs (blue), bulk Mn-PB (red) and PVP (green) were measured and are shown as in Figure 2(c). The IR spectra of the PVP coated Mn-PB NPs and the uncoated Mn-PB both show characteristic C≡N stretching vibrations at ~2073 cm^−1^ and 2082 cm^−1^ [not appearing as separate peaks in Figure 2(c) due to the wide plotting range to show all the features]. The IR spectrum for Mn-PB NPs shows additional bands at 1650 cm^−1^, 1421 cm^−1^ and 1280 cm^−1^, which overlap with the bands for PVP. This and the TEM photo in Figure 2(b) show that PVP is covering the Mn-PB NPs’ surface. The presence of water is also confirmed by the peak at 3465 cm^−1^ (O-H stretching) and is consistent with the fact that PVP is hydrophilic. The thickness for the PVP-coating is estimated less than 10 nm, which can let at least 98% of the soft X-ray (> 630 eV) pass through its layer and reach the Mn-PB NPs samples. For NRVS, the transmission of X-ray at 14.4 keV of the 10 nm PVP is close to 100%.

The structural analogs, KGd[Fe(CN)_6_] and KEu[Fe(CN)_6_], were synthecized with the same procedure described in SI.2 [19, 20] but by introducing Eu, Gd sources (instead of Mn source) to the K_4_[Fe(CN)_6_] complex. The ^57^Fe enriched K^57^Fe[Co(CN) _6_] was produced with a similar procedure by introducing ^57^Fe source (e.g. ^57^FeCl_2_) to the K_3_[Co(CN)_6_] complex alternatively. The NRVS spectrum for (NH_4_)_2_Mg[^57^Fe(CN)_6_] was directed cited from the previous publications [22, 28]. The comparison samples in this study, MnF_2_, K_3_[Co(CN)_6_], FeO, K_4_[Fe(CN)_6_], and K_3_[Fe(CN)_6_], were purchased from commercial sources and used without further process. Additional energy calibration samples, Fe_2_O_3_, and CoO, were also purchased from commercial sources and used without further process.

For better control of their surface condition, all the samples, include the main sample and all the comparison, reference and energy calibration samples were stored inside an N_2_ based glovebox for over 7 days before loading into the measurement chamber for L XAS measurement. For NRVS measurement, this step becomes unnecessary.

## 3. Results and Discussions

In general, oxidation state is one of the most-pursued chemical properties. Resolved oxidation states or electron/charge densities have helped the understanding of many chemical processes [36, 70], while an unresolved oxidation state has contributed to many longstanding controversies in history [71–76]. This is especially true for transitional metal complexes [36, 70]. L-edge XAS for 3d metals which measures the 2p → 3d transition is one of the best methods to investigate the oxidation states and electronic spin state via L_3_ absorption edge positions or their centroid energies. For example, L XAS exhibits about 2 eV change in their L_3_ centroids per oxidation state change (eV/oxi) for the Mn complexes and 0.9 eV/oxi for Ni complexes [29, 36, 77]. The ls-Ni(II) also has a higher L_3_ centroid than the hs-Ni(II). For Mn complexes, the branching ratio of L_3_/(L_3_+L_2_) sometimes also has been used to determine the Mn oxidation states, where the L_3_ and L_2_ mean the integrated intensities at the L_3_ and L_2_ edges respectively [36, 77]. These L-edge centroids are often sufficient to assign the oxidation states.

The spectral multiplets are more essential to identify metal’s oxidation states, electronic spin states and its coordination symmetries [36, 38, 77–80]. The match of their spectral multiplets in two spectra indicates that the two samples have the same metal sites (or at least extremely similar metal sites). Due to its fingerprint-like multiplets, L-edge XAS is sensitive to and has been widely used to identify the oxidation states, spin states and other information mentioned above for metal centres in various 3d transition metal complexes and metalloenzymes [36, 38]. For example, it has been used to identify Ni(I, II, III) and even Ni(II) with different electronic spin state (hs vs. ls) successfully [37–39].

### 3.1 Manganese L-edge XAS

**Figure 3.**
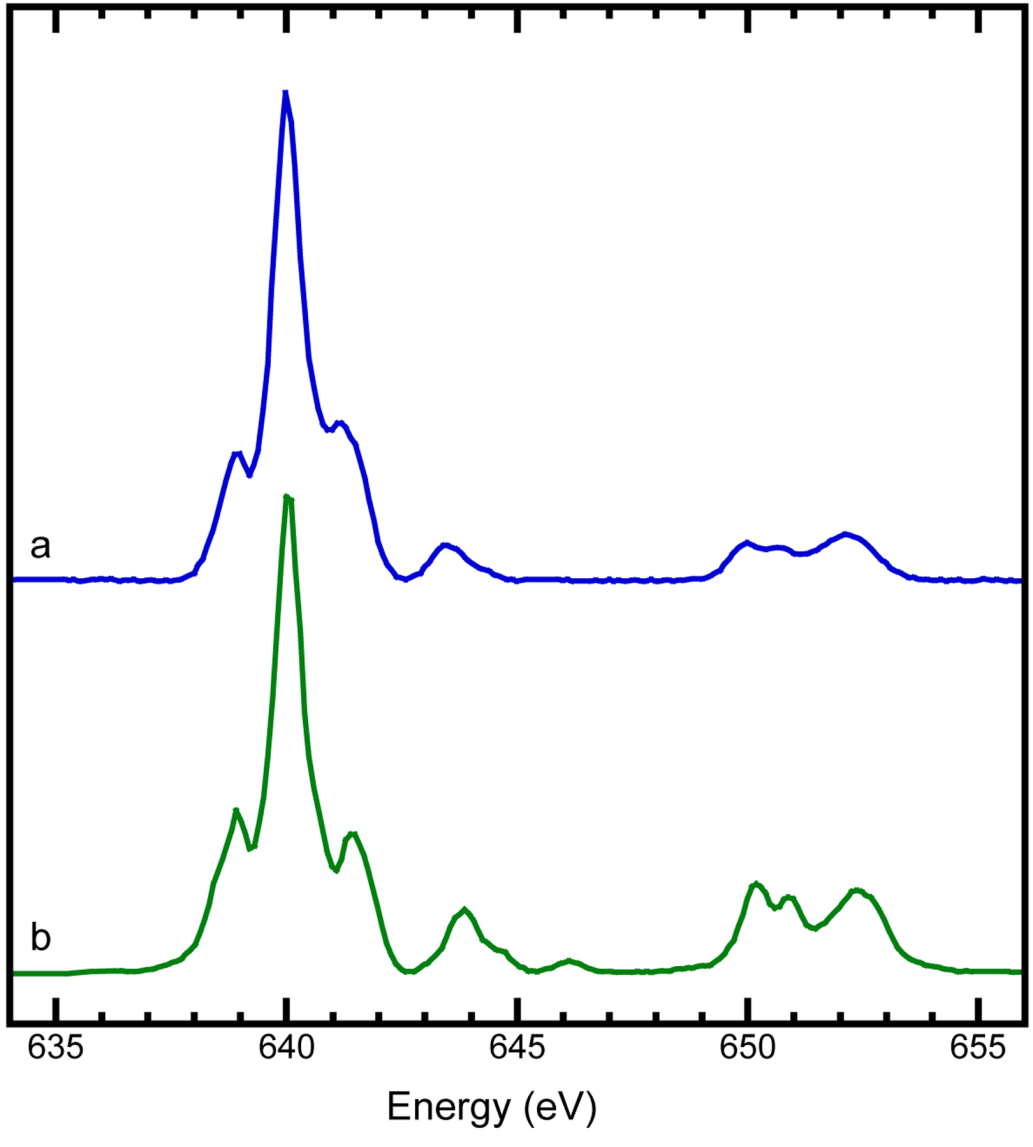
Mn L-edge XAS for K_2_Mn[Fe(CN)_6_] (**a**) and MnF_2_ (**b**).

The Mn L-edge XAS for our nanoparticulated K_2_Mn[Fe(CN)_6_] is shown as in Figure 3(a). It shows an almost identical multiplet feature in both L_3_ and L_2_ regions as the one for MnF_2_ [Figure 3(b)]. The latter is well known to have a hs-Mn(II) center [81], making our assignment of the Mn center in K_2_Mn[Fe(CN)_6_] as a hs-Mn(II) rather reliable. In comparison with the Mn L-edge XAS for various other complexes with various Mn oxidation states and spin states in Figure S2 and Figure S3 in SI.3, our Mn L-edge XAS for K_2_Mn[Fe(CN)_6_] [Figure 3(a)] is similar to that of the MnO (Figure S2, the bottom curve in the left panel) [78, 82] and MnF_2_ (Figure S2, the second curve from the top in the right panel) [36, 81] which have a hs-Mn(II). On the other hand, it is very different from those of the other Mn complexes with oxidation states ranging from +3 to +7 (Figure S2, left panel) [36, 78] or from more covalent Mn(II) complexes (Figure S2, right panel) [81]. In comparison with Figure 3(a) (oxidation state = +2), most of the L-edge XAS in the left panel of Figure S2 (oxidation state = +3 to +7) have higher L_3_ peak centroids, reflecting the correlation between the oxidation state and the L_3_ centroid. These observations are consistent with the discussion in the associated reference [36, 78, 79, 81].

According to a previous publication [81] and as re-illustrated in Figure S3, the ls-Mn(II) in [Mn(CN)_6_]^4−^ ion[81] shows a sharp peak at 638.6 eV which is well separated from the rest of the L_3_ multiplets. This is a quite unique feature for a [Mn(CN)_6_]^4−^ [81]. The fact that K_2_Mn[Fe(CN)_6_] does not have this feature (especially the lack of a “pre-L_3_” peak at 638.6 eV) indicates the Mn(II) in K_2_Mn[Fe(CN)_6_] is not in the C-bound position as that in the [Mn(CN)_6_]^4−^ ion. Although there is no particular L XAS report found for [Mn(CN)_6_]^3−^, previous reports revealed that there are about 2 eV L_3_ centroid shift between Mn(II) and Mn(III) [36, 83], excluding the possibility of a [Mn(CN)_6_]^3−^ site as well. Taken as a whole, our L XAS data show that the complexed Mn is not in the C-bound position in 6CN^−^, or in other words, it could occupy the position of the N-bound hs-Fe(III) site in PB (Figure S1). This in turn implies that Fe in K_2_Mn[Fe(CN)_6_] should remain in the C-bound position in the [Fe(CN)_6_]^4−^ ion

### 3.2 Cobalt L-edge XAS

**Figure 4.**
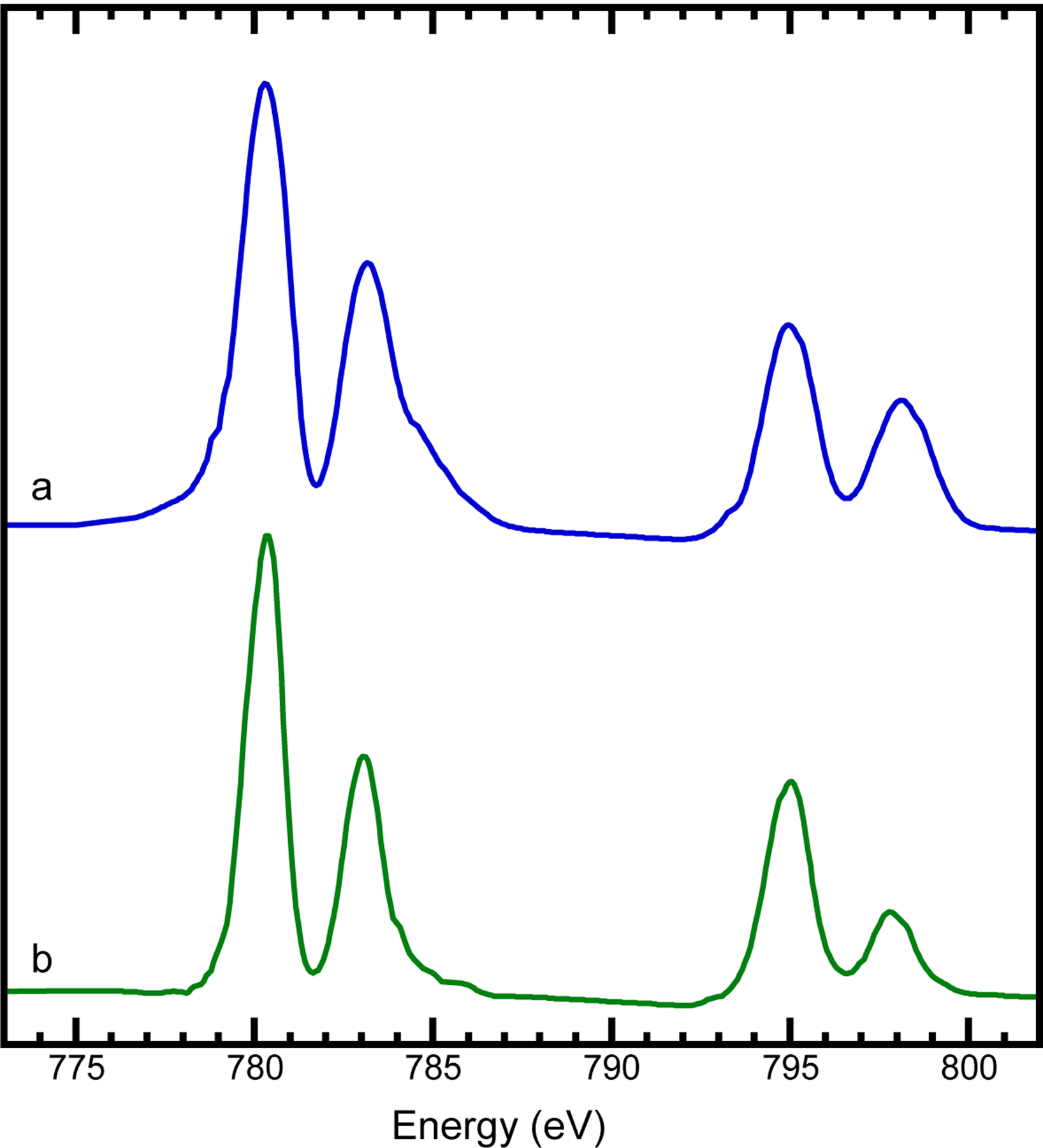
Co L-edge XAS for K^57^Fe(II)Co(III)(CN)_6_ (**a**) and K_3_Co(III)(CN)_6_ (**b**).

K_2_Mn[Fe(CN)_6_] was prepared by introducing a Mn(II) source (i.e,. MnCl_2_) into K_4_Fe(CN)_6_ to form a PB analog compound (SI.2) [19, 20]. According to L XAS [Figure 3(a) vs. Figure 3(b), Figure S2, and Figure S3], Mn is found to be in the N-bound position in PB (Figure S1). In an “opposite” approach, an ^57^Fe(II) source (i.e,. ^57^FeCl_2_) was complexed into the [Co(CN)_6_]^3−^ ion to form an alternative PB analog compound of K^57^Fe[Co(CN)_6_] where Co should remain in the C-bound position in the [Co(CN)_6_]^3−^ ion. Therefore, Co vs. Mn L XAS can provide a meaningful comparison insight into the two different cases. For this purpose, Figure 4 shows the Co L XAS for the comparison samples K^57^Fe[Co(CN)_6_][Figure 4(a)] and the reference sample K_3_[Co(CN)_6_][Figure 4(b)].

First, Figure 4(b) satisfactorily reproduced the previously reported results on the same complex (Figure S4 left panel, the bottom curve) [84, 85], establishing a good reference spectrum for the ls-Co(III) in [Co(CN)_6_]^3−^. The fact that Co L XAS spectrum for KFe[Co(CN)_6_][Figure 4(a)] has an overall resemblance as well as the same characteristic feature for the ls-Co(III) in K_3_[Co(CN)_6_] [Figure 4(b)] shows that the Co in KFe[Co(CN)_6_] is in the C-bound position in [Co(CN)_6_]^3−^ [84]. There are some minor differences in the intensity ratio and peak width, but the overall feature resembles each other pretty well. We noticed that our spectrum for K_3_[Co(CN)_6_] [Figure 4(b)] also has a slight difference in peak positions in comparison with the previous reports (Figure S4, left panel, the bottom curve) [84], probably due to the minor difference in energy calibrations.

On the other hand, when comparing with L XAS reports for other Co complexes in Figure S4 [84–89], our Co L XAS for KFe[Co(CN)_6_] appears to be very different from them. In particular, the Co L XAS for KFe[Co(CN)_6_] has a very different multiplet from the L XAS for the Co in the N-bound position in other PB analogs, such as in ACo[Fe(CN)_6_].xH_2_O (A = Na or K) (the left panel of Figure S4, top two curves). This rules out the possibility that the Co in KFe[Co(CN)_6_] is in the N-bound position to the [Fe(CN)_6_]^4−^ ion. In addition, its spectroscopic feature is also very different from the Co L XAS features of various Co(II), ls-Co(III) and hs-Co(III) complexes (Figure S4 left and right panels) [84–89]. In short, the L XAS for KFe[Co(CN)_6_] in Figure 4(a) only resembles the L-edge XAS of a ls-Co(III) in a [Co(CN)_6_]^3−^ ion [e.g. Figure 4(b) or the reported spectrum in Figure S4 (the bottom curve at the left panel)]. The Co(III) in a strong ligand field created from the 6CN^−^ ions has to be a ls-Co(III) instead of a hs-Co(III). This observation is not unexpected, just like the Fe is a ls-Fe(II) in K_2_Mn[Fe(CN)_6_].

### 3.3 Iron L-edge XAS

Sections 3.1 and 3.2 present a comparison of L XAS features between Mn and Co – the former is in the N-bound position while the latter is in the C-bound position. This in turn puts their Fe in different positions in the PB structure and the two complexes should have different Fe L XAS spectra.

**Figure 5.**
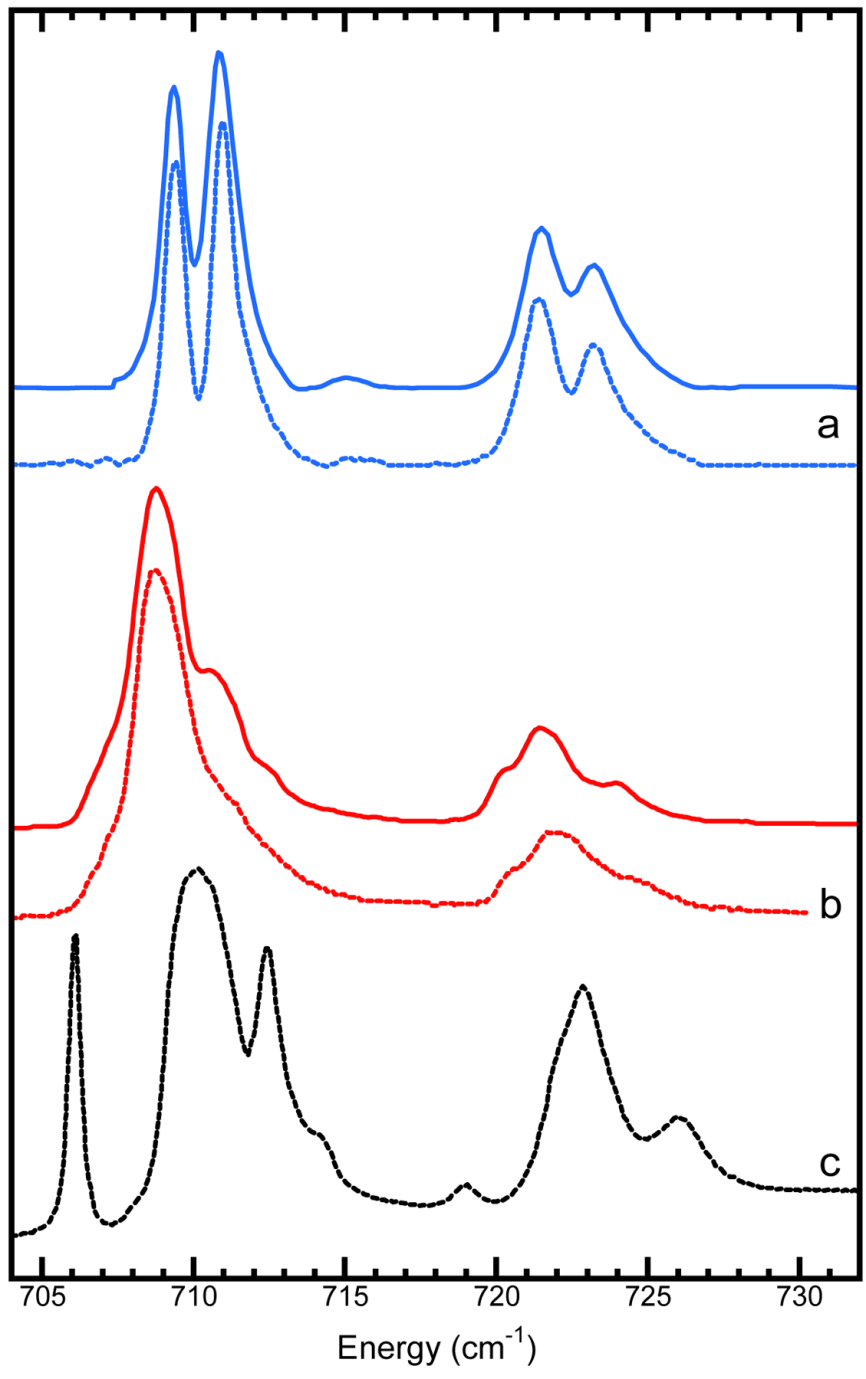
**Fe L-edge XAS for K**_2_**Mn[Fe(CN)**_6_**] (a, solid line), K**_4_**[Fe(CN)**_6_**] (a, dashed line), KFe[Co(CN)**_6_**] (b, solid line), FeO (b, dashed line) and K**_3_**Fe(III)(CN)**_6_ **which has a sharp characteristic peak at 706 eV (c). The complexes in (a) have a ls-Fe(II), the ones in (b) have a hs-Fe(II), the one in (c) has a ls-Fe(III).**

In this section, the Fe L XAS of K_2_Mn[Fe(CN)_6_] [Figure 5(a), solid line] and its comparison complex KFe[Co(CN)_6_] [Figure 5(b), solid line] are evaluated. K_2_Mn[Fe(CN)_6_] shows a typical spectral feature of a ls-Fe(II) especially when it is compared with that of the ls-Fe(II) in K_4_Fe(CN)_6_ [Figure 5(a), dashed line, or Figure S5, the left panel (a) – dark line][90]. This spectral feature is also similar to those of several other ls-Fe(II) complexes cited in Figure S5 [the right panel (c)–(e)] [91]. There is no surprise because these cited complexes all have CO or CO/CN ligands, while both CO and CN are very similar strong ligands [92–94]. On the other hand, the Fe L XAS of K_2_Mn[Fe(CN)_6_] [Figure 5(a), solid line] does not have any similarity with the Fe(0) in (NEt4)_2_Fe^0^(CO)_3_(CN)_2_ or / Fe(I) in Fe^I^(pdt)(PMe3)(CN)(CO)4 in Figure S5 [the right panel (a)–(b)] [91] or Fe(III) in other references not cited in Figure S5, including FeO(OH)_2_ [95], Fe_2_O_3_ [96] FeF_3_ [96], FeNO_3_ [97]. In particular, the Fe L XAS for the ls-Fe(III) in K_3_[Fe(CN)_6_] [Figure 5(c)] has a prominent sharp peak at ~706 eV, while Fe(II) in K_4_[Fe(CN)_6_] or K_2_Mn[Fe(CN)_6_] [Figure 5(a)] does not have this feature. This stand-alone peak is the major characteristic feature of the d^5^ ls-Fe(III) [90, 98] – the only difference among different d^5^ ls-Fe(III) complexes is how far this peak is from the highest L_3_ peak. In addition to the re-measurement [Figure 5(c)], a published L XAS spectrum for K_3_[Fe(CN)_6_] is also cited from reference [90] and re-presented in Figure S5 [the left panel (b) – dark line]. From the comprehensive comparison, K_2_Mn[Fe(CN)_6_] certainly has a ls-Fe(II) feature rather than a ls-Fe(III) feature [98].

Figure 5(b) shows a hs-Fe(II) in the N-bound position connecting to the [Fe(CN)_6_]^4−^ ion, in contrast to the ls-Fe(II) in [Fe(CN)_6_]^4−^ shown in Figure 5(a) or Figure S5 - the left panel (a). We, therefore, assign the comparison complex KFe[Co(CN)_6_] to have a hs-Fe(II) in the N-bound position.

Comparison between the Mn/Fe L-edge XAS for K_2_Mn[Fe(CN)_6_] vs. Fe/Co L-edge XAS for KFe[Co(CN)_6_] provides the readers with a clear understanding and an overall picture about the electronic properties for the Mn, Fe, and Co. Specifically, the ls-Fe(II) in C-bound position [Figure 5(a)] is consistent with the hs-Mn(II) in the N-bound position [Figure 3(a)] for K_2_Mn(II)[Fe(II)(CN)_6_] and the hs-Fe(II) in the N-bound position [Figure 5(b)] is consistent with the ls-Co(III) in the C-bound position [Figure 4(a)] for KFe(II)[Co(CN)_6_].

Photoreduction in SR beam is often an issue for L-edge XAS measurements because the beam intensity is high, while the soft X-ray penetration depth is very small [99]. For example, Colison et al. [98] found that K_3_[Fe(CN)_6_] or [Co (acac)_3_] can be photo reduced from Fe(III) to Fe(II) species in 26 mins or from Co(III) to Co(II) species in 20 mins. To ensure that the Mn(II), Co(III) and Fe(II) in our samples were not produced from the photoreduction (or oxidation), we used a heavily defocused beam of 1×1 mm^2^ in the beam size to greatly reduce the beam brightness and thus the photo-reduction rate (by 2–3 orders) in order to prevent or at least minimize such problem. In addition, a reduced beam flux was also obtained by setting much narrower beam slits as described in detail in the experimental section. To further evaluate the issue, we also quickly scanned on fresh points and frequently change the measurement spots using a beam size of 0.5×0.5 mm^2^ and 3 time increased beam flux (and thus 12 time increased beam brightness). No photoreduction has been found for the complexes subjected to this testing condition, let alone under the real measurement condition. For example, we did not see any Fe(III), Mn(III) or Co(II) even in the first 1 minute of each rapid scan on fresh points under the increased beam brightness. We did perform a few series of repeated testing scans in several spots and did not find any change in the spectral features during the whole time periods of over 3–5 hours each. Nevertheless, all the L-edge XAS spectra presented in this study are only from the individual scans in fresh points.

In short, the comprehensive L XAS analysis on Mn, Co, Fe inside K_2_Mn[Fe(CN)_6_] as well as its comparison complex KFe[Co(CN)_6_] provides the site specific information on the oxidation states and spin states for these metals, which is critical in evaluating the new, non-gadolinium MRI contrast agents. In particular, our L XAS indicates that K_2_Mn[Fe(CN)_6_] contains a hs-Mn(II) and an ls-Fe(II), leading to a total spin per molecule of 5/2. This is suitable for an MRI agent.

### 3.4 ^57^Fe NRVS

Since L-edge XAS and IR measurements are surface sensitive methods, bulky sensitive ^57^Fe-specific NRVS is an excellent addition to evaluate the Fe information in various PB-like complexes. Similar to other vibrational spectroscopies, NRVS is sensitive to the oxidation states and has been widely used to assign the oxidation states for ^57^Fe sites in different complexes and enzymes [28, 48, 49, 54]. It also provides the ligand identity and symmetry around the ^57^Fe site(s) [63, 64]. For PB or PB-like compounds, NRVS can also indicate whether the spectral feature is from the Fe in the C-bound position in [Fe(CN)_6_]^4−^ or from the off-sphere N bound position [22, 28].

**Figure 6.**
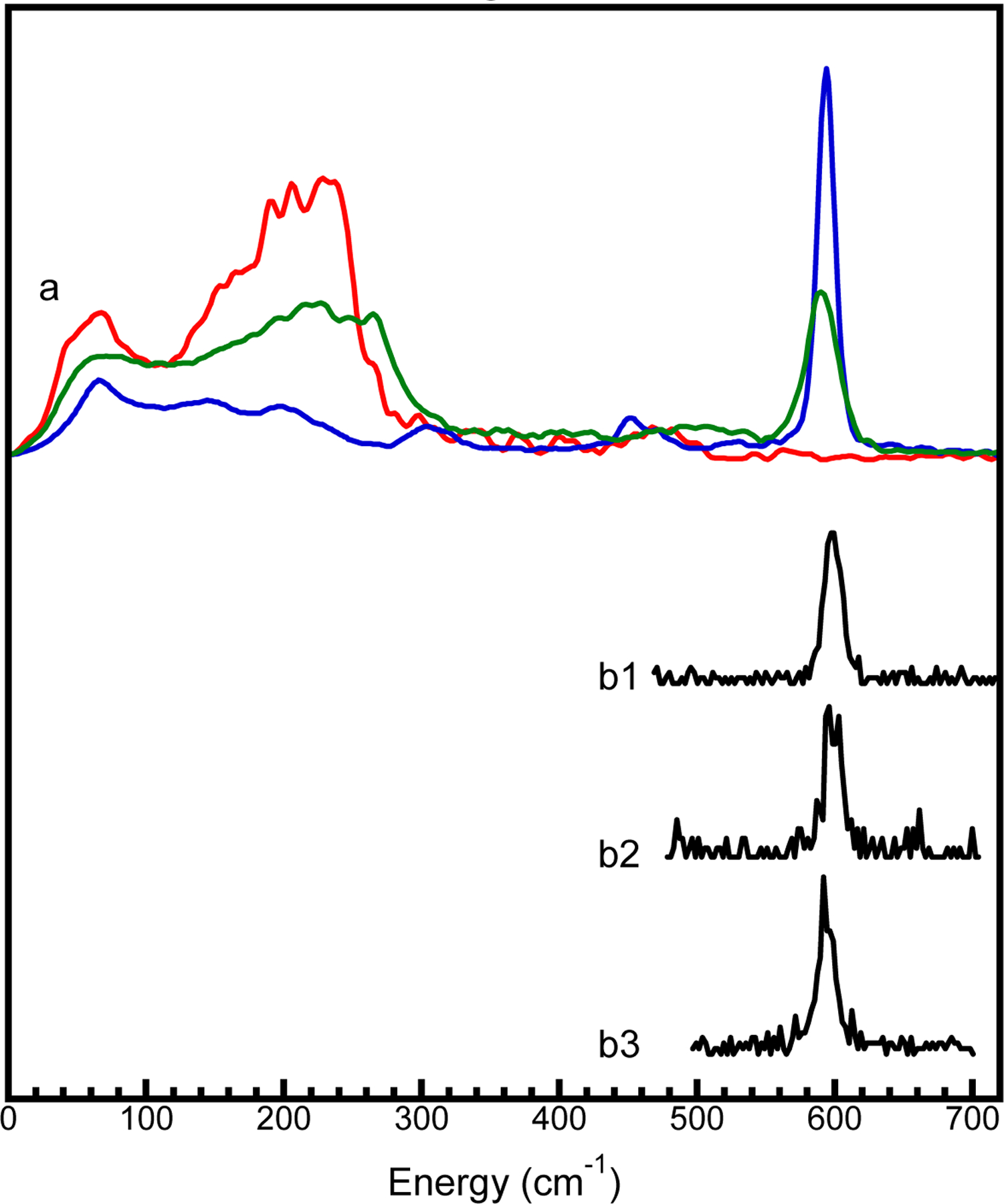
**(a)** NRVS spectra for ^57^Fe enriched PB (green), (NH_4_)_2_Mg(II)- [^57^Fe(II)(CN)_6_] [44] (blue) and K^57^Fe(II)[Co(III)(CN)_6_]; (b1-b3) skip scan measured regional NRVS peak for K_2_Mn(II)[Fe(II)(CN)_6_] (b1), KEu(III)[Fe(II)(CN)_6_] (b2) and KGd(III)[Fe(II)(CN)_6_] (b3).

The PB NRVS has two major peaks [Figure 6(a), green] [22, 28]: 1) the strong interactions between the ls-Fe(II) and 6 CN^−^ ions, which leads to a sharp peak at 594 cm^−1^; and 2) the weaker interaction between the off-sphere hs-Fe(III) and the [Fe(CN)_6_]^4−^ ion (via Fe…N interactions), which produces an additional broad hump at 120 – 280 cm^−1^. Please note that the sharp peak position was mentioned in several previous publications [28, 44] at 602 cm^−1^, but it appears at 594 cm^−1^ [22] with the calibration method used in our research team (380 cm^−1^ for the Fe-Cl stretch peak in (Et_4_N)[FeCl_4_] [28, 55]). Although we realize that there are different energy calibration standards employed by different research teams with no effort made thus far to unify the calibration standards among different teams, using one standard in one research team is meaningful.

To further illustrate the origin of each peak (or hump), either the C-bound ls-Fe(II) or the N-bound hs-Fe(III) in PB can be “substituted” with another metal, leaving only one site labelled with ^57^Fe. This approach creates two site–specific complexes: one is our comparison sample K^57^Fe[Co(CN)_6_]. Its NRVS has no peak at or around 594 cm^−1^ at all but has a lower energy hump at 120 – 280 cm^−1^ [the red curve in Figure 6(a)]. Another complex is (NH_4_)_2_Mg(II)[Fe(II)(CN)_6_] [44] with a [^57^Fe(CN)_6_]^4−^ ion that is weakly bound to Mg. This complex has one strong NRVS peak at 594 cm^−1^ (the blue curve) but almost no hump at 120 – 280 cm^−1^. These NRVS spectra well-illustrate how NRVS feature(s) can be used to confidently answer the question about the locations of their Fe sites in the corresponding PB analog compounds, i.e., in the C-bound position or the N-bound position.

Since K_2_Mn[Fe(CN)_6_] is not ^57^Fe labelled, its NRVS thus has a very weak signal (2% ^57^Fe in natural abundance sample vs. 100% ^57^Fe in the enriched samples). Therefore, measuring a full NRVS scan is out of the question. We instead only scanned a narrow region around the nuclear resonant peak at E_vib_=0 (±40 cm^−1^) and that around the sharp peak at ~ 594 cm^−1^ (e.g. 380–700 cm^−1^ – the presentation range here is a bit less) [22, 28]. Although the exact peak position for this sharp peak cannot be precisely located due to the skip scan operation (jumping from the nuclear resonant peak region to the targeted sharp peak region) [22, 28], the obtained peak feature is sharp and clear. The energy positions are less than ±1 meV from the peak position at 594 cm^−1^ [Figure 6(b1-b3) vs. 6(a)], while the skip scan uncertainty can be as large as 1.5 meV. This indicates the presence of a C-bound ls-Fe(II) in [Fe(CN)_6_]^4−^, confirming the assignment based on the L-edge XAS measurements. This shows that a C-bound ls-Fe(II) is true for the sample’s bulk of K_2_Mn[Fe(CN)_6_], not just on its surface (L XAS).

Previously measured partial NRVS spectra for two unenriched samples KEu[Fe(CN)_6_] and KGd[Fe(CN)_6_] are also added to Figure 6 (b2 and b3) for comparison – both of them also have a clear feature for a characteristic peak for the C-bound ls-Fe(II) in [Fe(CN)_6_]^4−^ (around 594 cm^−1^). On the other hand, the lack of sharp NRVS peak at or around ~ 594 cm^−1^ shows that the comparison complex K^57^Fe[Co(CN)_6_] does not have a C-bound ls-Fe(II). The NRVS comparison further indicates that the complexed element stays in the N-bound position, which is consistent with the Mn, Co and Fe L XAS results. For reference, in a previous publication [22], ^151^Eu and ^57^Fe NRVS were used to measure KEu[Fe(CN)_6_] vs. Eu[Fe(CN)_6_] and to conclude the complexed metal (Eu) remains in the N-bound position in PB analogs.

In addition, NRVS can also identify whether the Fe is an Fe(II) or an Fe(III) [22]. In particular, as shown in Figure S6 in SI.6 [22], the Fe(III)-(CN)_6_ interaction has a much lower energy position (at 516 cm^−1^) and a much wider peak (32 cm^−1^ in FWHM) than the Fe(II)-(CN)_6_ does (at 594 cm^−1^ with a 14 cm^−1^ FWHM). In general, this trend is not surprise for complexes with CN or CO ligands [90, 93, 94, 100, 101]. In comparison, K_2_Mn[Fe(CN)_6_]) indeed has a ls-Fe(II), not a ls-Fe(III).

## 4. Conclusions

In this publication, we have conducted detailed measurements of L-edge XAS on Mn and Fe, respectively, for the nanoparticulate PB analog K_2_Mn[Fe(CN)_6_] that has been synthesized and evaluated as a potential MRI agent [19, 24]. The results obtained from such L XAS experiments allow us to unambiguously conclude that K_2_Mn[Fe(CN)_6_] has a hs-Mn(II) bound to N and a ls-Fe(II) bound to C in the PB structure. For comparison, Co and Fe L XAS on KFeCo[(CN)_6_] show that it has a hs-Fe(II) surrounded by N and a ls-Co(III) surrounded by C in the PB structure. Accordingly, K^+^ ions have to be incorporated into the structure to maintain electroneutrality of the overcall formula and a ratio of Mn: Fe=1:1 or Fe:Co=1:1 for the corresponding complexes.

To rule out the possible issues of the surface effect, the bulk-sensitive ^57^Fe NRVS study is also performed for K_2_Mn[Fe(CN)_6_] and its reference samples KEu[Fe(CN)_6_] and KGd[Fe(CN)_6_]. The NRVS results are consistent with the above conclusions obtained from the surface sensitive L XAS.

In addition to the evaluation of its relaxvity properties of K_2_Mn[Fe(CN)_6_] to assess its suitability as a prime candidate for the next-generation non-gadolinium MRI agent, the current studies well illustrate the many significant advantages of using L XAS and NRVS in combination on the same sample in revealing the site-specific information for nanoparticulate PB analog complexes or other complexes in general.

## 5. Future work

In the future, it will be interesting to observe whether the so-called coordination isomers of this or similar complex(es) or certain types of solid-solutions where Mn(II) and Fe(II) ions (or other pairs of metals) are partitioned between the C-bound and the N-bound positions in PB analogs can be prepared if one uses a one-pot self-assembly synthesis involving the stoichiometric amounts of Fe(II), Mn(II), and CN^−^.

## Supplementary Material

Supporting-Information
