# Supplementary material for: X-ray and nuclear spectroscopies to reveal the element specific oxidation states and electronic spin states for nanoparticulated manganese cyanidoferrates and analogs
Source: Physchem. Author manuscript; Available in PMC 2025 Apr 30. (PMC12043228; doi:10.3390/physchem4010003)
Supplement: Supporting-Information [file NIHMS2053388-supplement-Supporting-Information.pdf]

X-ray and nuclear spectroscopies to reveal the element specific oxidation states and electronic spin states for nanoparticulated manganese cyanidoferrates and analogs

Hongxin Wang,[1,*] Songping D. Huang,[2,*] Anthony T. Young,[3] Stephen P. Cramer,[1,] Yoshitaka Yoda,[4] and Lei Li[5,†]

[1] SETI Institute, Mountain View, CA 94043, United States
[2] Department of Chemistry and Biochemistry, Kent State University, Kent, OH 44242, United States
[3] Lawrence Berkeley National Laboratory, Berkeley, CA 94720
[4] Precision Spectroscopy Division, SPring-8/JASRI, Sayo, Hyogo 679-5198, Japan
[5] Synchrotron Radiation Research Center, Hyogo Science and Technology Association, Kouto, Hyogo, 679-5165, Japan
[†] Current address: Institute of Advanced Science Facilities, Shenzhen, China

* Correspondence: HW hongxin.ucd@gmail.com, SH shuang1@kent.edu

Summary of Sample Preparation Procedures

Synthesis of $K_2Mn[Fe(CN)_6]$: First, 0.222 g of PVP (Average MW=40000) was dissolved in 20 ml of $K_3[Fe(CN)_6]$ (1 mM) solution. Then 20 ml of 1 mM $MnCl_2$ solution was added dropwise to the above mixture under vigorous stirring. A pale yellow dispersion formed immediately. The reaction mixture was then stirred for another ca. 3 hrs. The reaction product was dialyzed using regenerated cellulose tubular membrane (MWCO is 3.5kDa) against distilled water for 2 days. PVP-coated nanoparticles, denoted as MnPB NPs. Bulk MnPB were prepared by the same method, but without the addition of PVP into the solution. Briefly, 110 mg of $K_3[Fe(CN)_6]$ in 10 mL of deionized water was added to the aqueous solution (10 mL) of $MnCl_2.4H_2O$ (66 mg) while stirring. The precipitate was separated by centrifugation.

Synthesis of KFe(II)[Co(III)(CN)$_6$]: an aqueous solution of $FeCl_2 \cdot 4H_2O$ (1 mM, 15 mL) was added dropwise to an aqueous solution of K_3[Co(III)(CN)$_6$] (1 mM, 10 mL) under vigorous stirring. An off-white precipitate was formed slowly, and the reaction was stirred at room temperature for 8 hours before the precipitate was filtered and washed with deionized water and acetone three times each, respectively, to afford a white microcrystalline powder.

Synthesis of K^{57}Fe(II)[Co(III)(CN)$_6$]: a sample of ^{57}Fe powder was first dissolved in 2 mL concentrated HCl in a small beaker at 50 °C to give a colorless solution. The excess HCl was evaporated at this temperate for 6 hours to dryness before 15 mL deionized water was added to make an aqueous solution of $FeCl_2$ (1 mM). This colorless solution was added dropwise to an aqueous solution of K_3[Co(III)(CN)$_6$] (1 mM, 10 mL) under vigorous stirring. An off-white precipitate was formed slowly, and the reaction was stirred at room temperature for 8 hours before the precipitate was filtered and washed with deionized water and acetone three times each, respectively, to afford a white microcrystalline powder.
